# Incarcerated Littre’s Umbilical Hernia: A Case Report

**DOI:** 10.31729/jnma.8443

**Published:** 2024-02-29

**Authors:** Binod Bade Shrestha, Anmol Lamichhane, Rahav Chandra Pokhrel, Pratik Parajuli, Prince Goyal

**Affiliations:** 1 Department of Surgery, Manipal College of Medical Sciences, Pokhara, Kaski, Nepal; 2 Manipal College of Medical Sciences, Pokhara, Kaski, Nepal

**Keywords:** *case reports*, *hernia*, *Meckel diverticulum*

## Abstract

Littre's hernia is an extremely rare type of hernia which has Meckel's diverticulum as its content. A 63-year-old male, presented to the emergency department with chief complaints of swelling and pain around the umbilicus. The patient was diagnosed with an incarcerated umbilical hernia. Following the emergency laparotomy, the intraoperative finding depicted an umbilical Littre's hernia. The patient underwent open Meckel's diverticulectomy with mesh repair. Preoperative diagnosis of Littre's hernia is unlikely due to its low incidence and lack of specific radiological and clinical findings, but the role of computed tomography scan and ultrasound are important in differentiating between strangulated or incarcerated bowel and omentum and in guiding the urgency of operative management.

## INTRODUCTION

Meckel's diverticulum (MD) is the most common congenital anomaly of the gastrointestinal tract, but the occurrence of a Littre's hernia (LH) is an extremely rare condition.^1^ Adult Littre's hernia is rare and a systematic review confirmed only 53 cases. In that period, femoral hernia was most frequently reported (21 cases, 39.6%), followed by inguinal hernia (18 cases, 34.0%).^2^ The umbilical hernia was very rare, with only 6 cases (11.3%).^2^ Preoperative diagnosis of LH is unlikely due to its low incidence and lack of specific radiological and clinical findings.^1^ Rarely, it may undergo incarceration or strangulation, necrosis, and perforation. Repair of Littre hernia consists of resection of the diverticulum and herniorrhaphy or hernioplasty.^3^

## CASE REPORT

**Figure 1:**
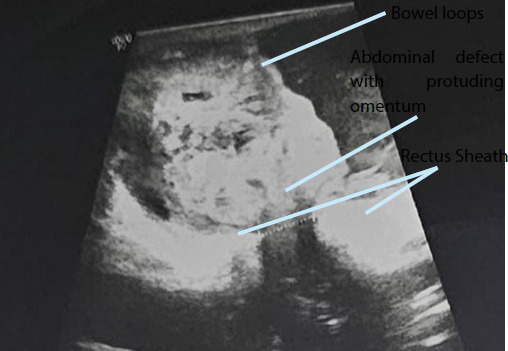
Ultrasonographic image showing a defect in the umbilical region with herniated bowel loops.

A 63-year-old male, a diagnosed case of alcoholic liver disease (ALD) spectrum with moderate ascites and portal gastropathy presented to the emergency department of tertiary care centre with swelling around the umbilicus for 19 months but for the last 3 days, the swelling was irreducible and was associated with pain and vomiting. On examination, there was an irreducible swelling of 5 × 3 cm^2^ with the presence of cough impulse. Ultrasonography (USG) abdomen and pelvis showed a defect of 9.8 mm noted in the umbilical region with herniation of bowel loops surrounded by free fluid and minimal internal vascularity which were features suggestive of obstructed umbilical hernia (Figure 1).

**Figure 2:**
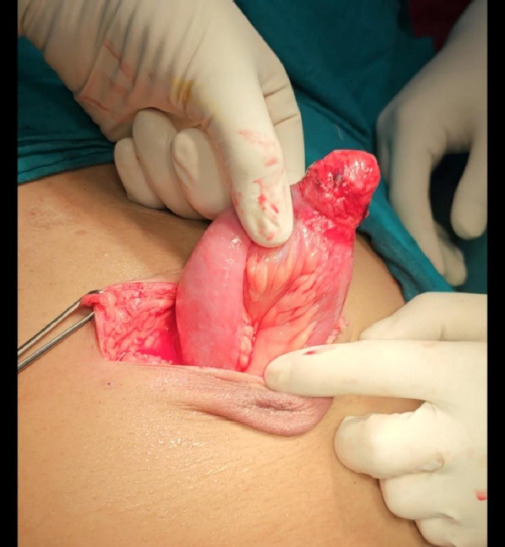
Intraoperative picture showing Meckel's diverticulum.

The patient was hence planned for emergency laparotomy. The patient was prepared pre-operatively with broad-spectrum antibiotics, was kept nill per oral (NPO) and all preoperative investigations were sent. Laboratory investigations revealed leucocytosis with neutrophilia with a white blood cell (WBC) count of 12,520 per cubic mm, coagulopathy with PT/INR of 24.7/2.06, anaemia with haemoglobin of 8.8 gm/dl and serum creatinine of 1.3 mg/dl. Intraoperatively, it was noted that it was incarcerated umbilical hernia containing Meckel's diverticulum with a defect of size 1.5 cm (Figure 2).

Meckel's diverticulum measured 5 cm in length and 2 cm in width with the base present in the ileum. The patient also had gross ascites with 2 litres of ascetic fluid in the peritoneal cavity. Resection of Meckel's diverticulum was done by gastrointestinal anastomosis linear stapler 80 mm. Continuous running suture with polydioxanone (PDS) 3-0 was taken in a stapler line and an abdominal drain was placed in the pelvic cavity. The defect was closed with PDS number 1 and hernioplasty was performed.

On postoperative day (POD) 3, blood was noted in the drain bag. The patient's haemoglobin dropped to 8.4 gm/dl for which he was transfused with 3 pints of whole blood and 2 pints of whole blood on POD 4 after which his haemoglobin improved to 11.9 gm/dl. The patient was also started on tranexamic acid and terlipressin for gastrointestinal bleeding. The patient's renal function test progressively deteriorated (rise in urea and creatinine with each subsequent day postop) and ultimately led to hepatorenal syndrome. On POD 9 patient was intubated as SpO_2_ dropped to 60% and the patient started gasping. The patient was kept on a ventilator under synchronized controlled mechanical ventilation (SCMV) mode. The patient was declared dead on POD 10. The cause of death was septic shock due to hepatorenal syndrome.

## DISCUSSION

Any hernia sac with the presence of Meckel's diverticulum is known as a Littre hernia.^1^ A Meckel's diverticulum is a true small bowel diverticulum which arises from incomplete obliteration of the vitelline duct during the fifth week of foetal development. It occurs on the antimesenteric border of the ileum and is predominantly located up to 150 cm from the ileocecal valve. Intestinal obstruction is the most common complication of MD in adults. Other complications include gastrointestinal bleeding, inflammation, perforation and malignant degeneration. Only 10% of cases of Littre's hernia occur in the umbilical region which makes this case more rare than a typical Littre's Hernia.^2^ Surgery is the appropriate treatment for complicated LH.^3^

Preoperative diagnosis of LH is unlikely due to its low incidence and lack of specific radiological and clinical findings but the role of CT and ultrasound are important in differentiating between strangulated or incarcerated bowel and omentum and in guiding the urgency of operative management.^1^

In this case the patientpresentedwith vague abdominal pain and irreducible painful umbilical mass indicative of strangulation. Initial diagnosis of incarcerated umbilical hernia was made by ultrasound but a complete diagnosis of incarcerated umbilical Littre's hernia was made intraoperatively. Treatment of adult incarcerated umbilical hernia involves two procedures: early relief of the incarceration and closure of the hernial orifice.^2,3^ There appears to be no disagreement regarding techniques to relieve incarceration. Still, various approaches to closing the hernial orifice have been considered, such as laparoscopic or laparotomic methods, simple suture or mesh closure, and one-or two-stage operations, and no standard approach has yet been determined.^2^ If MD is long diverticulectomy should be performed, if MD is short or narrow-based, there is no palpable mass within, the same diverticulum may be managed by a simple wedge resection with a transverse closure of the remaining ileal defect. In this case, the decision was made to do an open laparotomy to resect Meckel's diverticulum with hernioplasty since the length of the diverticulum was long i.e. 1.5 cm. Resection and anastomosis of a segment of the ileum may be required if there is oedema or inflammation at the base of the diverticulum to prevent postoperative ileal stricture. Literature also suggests avoiding the use of mesh where the bowel was ischaemic or perforated, to prevent the risk of mesh infection.^3^ But in this case, since the base of the diverticulum was healthy we kept a mesh to close the defect and we were also able to avoid resection of bowel segments.

Because of his late presentation and comorbidity of ALD which led to hepatorenal syndrome the patient lost his life. Systematic reviews show an eight times increase in mortality in patients undergoing umbilical hernia repair with cirrhosis compared to those without cirrhosis.^5,11^ Furthermore among cirrhotic patients, mortality was higher after emergency versus elective repair and perioperative ascites control is considered the most important factor governing outcome.^5,8,10^ The limitation in this case was that we were not able to do perioperative ascites control as the patient presented with an emergent condition and required immediate surgery.
